# Promoting Bone Health in Layer Chickens from the Perspective of Mitochondrial Energy Metabolism in Osteoclasts

**DOI:** 10.3390/ani16132046

**Published:** 2026-07-03

**Authors:** Zhiyu Su, Shuo Tian, Ruilong Song, Zongping Liu, Xishuai Tong

**Affiliations:** 1 Joint International Research Laboratory of Agriculture and Agri-Product Safety of Ministry of Education of China, Institutes of Agricultural Science and Technology Development, College of Veterinary Medicine, Yangzhou University, Yangzhou 225009, China; 222001315@stu.yzu.edu.cn (Z.S.); mx120241068@stu.yzu.edu.cn (S.T.); rlsong@yzu.edu.cn (R.S.); liuzongping@yzu.edu.cn (Z.L.); 2 Jiangsu Co-Innovation Center for Prevention and Control of Important Animal Infectious Diseases and Zoonoses, Yangzhou University, Yangzhou 225009, China; 3 Jiangsu Interdisciplinary Center for Zoonoses and Biosafety, Yangzhou University, Yangzhou 225009, China

**Keywords:** layer chickens, osteoclasts (OCs), mitochondria, bone metabolism

## Abstract

Layer chickens have dual physiological demands for rapid growth and continuous egg production. The maintenance of skeletal homeostasis in layer chickens relies on the precise coordination among OCs, osteoblasts (OBs), and osteocytes. The imbalances in the supply of nutrients such as calcium (Ca) and phosphorus (P), as well as dysfunction of the “gut–bone” axis, can disrupt normal bone development in layer chickens, leading to bone diseases such as tibial dyschondroplasia (TD) and osteoporosis (OP), seriously damaging the production performance of layer chickens. This review systematically summarizes the knowledge background of the metabolic reprogramming of OCs in layer chickens, especially mitochondria-mediated biological processes, including oxidative phosphorylation (OXPHOS), glycolysis, reactive oxygen species (ROS) signaling, mitophagy, etc. Notably, the co-culture system of OCs derived from the bone marrow cavity of embryos in vitro has been established in laying chickens. However, there are few reports on the study of mitochondrial metabolism of OCs using this model. Therefore, this review particular focuses on the bone metabolism mediated by OCs in layer chickens and proposes future research priorities, including the application of gene editing and multi-omics methods to ultimately achieve targeted nutritional or pharmacological interventions for optimizing mitochondrial function and promoting bone health.

## Simple Summary

Bone health is essential to the rapid growth or the high egg production of layer chickens. However, influencing factors in layer chicken farming, such as poor nutrition, intestinal infections, and gut microbiota imbalance, can impair bone development, for example, by causing an increase in the number or activity of osteoclasts (OCs), which is one of the reasons for bone development imbalance. At the cellular level, OCs are responsible for degrading bone tissue, and their differentiation and maturity require large amounts of energy to function properly. This energy is mainly supplied by the mitochondria, the major powerhouses within OCs. When the mitochondrial function of OCs is impaired or overactive, differentiation, maturation, and bone resorption are adversely affected, leading to various bone diseases, such as leg defects, an important cause of flock culling and welfare losses. This review primarily focuses on the study of mitochondrial metabolism in layer chickens’ OCs, hoping to build a conceptual bridge toward layer chickens’ bone health. We identify critical knowledge gaps related to mitochondrial metabolism in layer chickens’ OCs and propose a translational research roadmap, aiming to guide future studies and ultimately provide new strategies for preventing bone disorders and improving layer chickens’ welfare.

## 1. Introduction

Bone diseases represent a major obstacle to the sustainable development of the modern layer chicken industry, imposing significant economic burden and animal welfare challenges in layer chicken production. Keel bone damage and OP are common bone health issues in layer chicken production. Li et al. [1] systematically elaborated on the impact of keel bone damage on the welfare and productivity of layer chickens, noting that it not only causes pain, stress, and inflammatory responses but also reduces the performance and egg quality of laying chickens. Similarly, there is a serious imbalance between rapid growth and bone development in broilers, leading to a widespread occurrence of leg diseases. Moreover, leg diseases in broilers restrict their locomotor activity, reduce their productive performance and meat quality, and severely compromise animal welfare due to pain and limping [2]. Collectively, bone health is a critical factor in ensuring the economic efficiency and animal welfare of poultry, especially layer chickens, warranting particular attention in the context of global intensive production.

However, the unprecedented pursuit of high egg production imposes extreme metabolic demands on the bone of layer chickens. Bone is a core organ for movement, support, and mineral metabolism in layer chickens, and its homeostasis directly affects growth performance and egg production efficiency. Correspondingly, in order to meet the demands of growth, development, and egg production, the bone of layer chickens is continuously mobilized and remodeled for dynamic balance, mainly achieved by OC-mediated bone resorption and OB-mediated bone formation. In bone remodeling, the bone matrix is dissolved or resorbed by acidic substances and various hydrolytic enzymes secreted by OCs, and the proper execution of these functions depends on the highly dynamic metabolic reprogramming of OCs [3,4]. The metabolic reprogramming of OCs, mainly encompassing shifts in energy metabolism and signaling pathways, enables these bone-resorbing cells to efficiently mobilize bone mineral stores. This reprogramming allows the rapid cyclic resorption of medullary bone in layer chickens, providing 2–3 g of Ca for eggshell formation and the activation of OCs preceding the rise of estradiol at sexual maturity [5]. Therefore, the metabolic reprogramming of OCs in layer chickens is crucial because it underpins high-efficiency mineral mobilization for egg production while mitigating the risk of metabolic bone diseases during rapid growth. Nevertheless, systematic investigations specifically focused on the metabolic reprogramming of OCs using poultry models remain scarce.

The differentiation of OCs mainly relies on mitochondrial OXPHOS to obtain energy, while mature OCs complete active bone resorption and rely on aerobic glycolysis and other related signaling molecules, such as receptor activator of nuclear factor-κB (RANK) ligand (RANKL), RANK, myelocytomatosis oncogene (MYC), estrogen-related receptor alpha (ERRα), peroxisome proliferator-activated receptor gamma coactivator 1-beta (PGC-1β), and hypoxia inducible factor-1 alpha (HIF-1α) [4,6]. In addition, mitochondrial ROS are involved in the differentiation and maturation of OCs, playing a dual role as an essential second messenger. However, excessive ROS production leads to enhanced OC activation, causing oxidative damage and inducing oxidative stress, promoting pathological bone resorption such as OP [7]. Importantly, mitophagy is essential to the differentiation and maturation of OCs by eliminating damaged mitochondria and maintains mitochondrial quality. Research has found that the coordinated operation of the “lysosome/ferrum (Fe)/mitochondria” axis provides stable energy support for bone resorption, while Fe overload triggers the “Fenton reaction” and amplifies oxidative stress, driving excessive activation of OCs and ultimately causing OP [8].

This review constructs a conceptual framework centered on mitochondrial energy metabolism of OCs in layer chickens, exploring the master regulator of the function of OCs. Importantly, previous studies have reported the establishment of co-culture systems of OCs in layer chickens and direct observation of the differentiation of OCs derived from bone marrow medulla in vitro [9,10,11]. Therefore, these existing model systems provide the technical foundation for the research agenda proposed in this review. We systematically synthesize current knowledge on bone development, cellular metabolism, and the metabolic reprogramming of OCs within this framework to address the lack of evidence on metabolic reprogramming and its role in mechanism-based bone diseases of OCs in layer chickens. Finally, we identify these gaps and propose future research priorities, including the application of gene editing and metabolomics of OCs in layer chickens, to discover scientific theories for preventing and treating skeletal diseases in commercial layer chickens.

## 2. Cellular Basis of Bone Remodeling in Layer Chickens

Bone development occurs via two conserved modes, endochondral and intramembranous bone differentiation, in layer chickens and vertebrates [12,13]. During sexual maturity in laying chickens, the remodeling of the tibial transcriptome and metabolome gradually increases, particularly by activating pathways such as cholesterol and phospholipid metabolism, as well as matrix vesicle biosynthesis, thereby promoting medullary bone formation and mineralization. This drives rapid transition from structural growth to medullary bone Ca supplying metabolism, preparing laying chickens for subsequent egg production, and the eggshells rapidly mineralize in laying chickens, which also requires P and other trace elements [5,14]. Ca deficiency leads to bone loss, manifested as decreased bone mass and strength of the femur and tibia, as well as reduced egg production and egg quality of layer chickens [15]. The content of P in the diet should neither be too high nor too low, and use of organic trace minerals (protein-based compounds) increases daily egg production and improves egg quality, including increasing eggshell thickness and strength [16].

Ossification occurs within the cartilaginous template of the endochondral bones, and the bone collar also originates and occurs within the surrounding fibroblast cartilage perichondral sheath, while intramembranous bone differentiation is formed by direct differentiation of OBs with mesenchyme [13]. Specially, OCs initially originated from the yolk sac are crucial during the bone development of embryos, while hematopoietic-derived osteoclasts play an important role after birth [17,18]. Osteoclasts are very active in the early stages of bone development, especially RANKL secreted by OBs, and osteocytes can induce the differentiation of OCs [19,20,21]. Correspondingly, OBs and osteocytes restrict the recruitment and activation of OCs by secreting or expressing osteoprotegerin (OPG) and also regulate the ratio of RANKL to OPG to monitor the differentiation and maturation of OCs [22,23]. Therefore, during the growth and the development of layer chickens, various bone cells continuously interact to remodel the skeleton and maintain dynamic homeostasis. Bone remodeling is the ongoing process of bone formation and bone resorption, primarily executed by osteocytes, OBs, and OCs [24]. This continuous bone remodeling is an energetically expensive process that involves the synthesis and secretion of bone matrix by OBs, especially the acidification and enzymatic degradation of bone matrix by OCs, and demands massive amounts of ATP. Therefore, the mitochondria of bone cells are not merely passive organelles but active decision makers in bone homeostasis.

Osteocytes, derived from OBs, are the most abundant cells in mature bone (90–95%), and they feature dendritic structures with long cytoplasmic processes that connect with other cells and blood vessels. OBs are mononuclear cells rich in organelles, essential to bone formation and regeneration, and they synthesize and secrete bone matrix proteins, such as type I collagen (COL1), alkaline phosphatase (ALP), bone sialoprotein (BSP), osteopontin (OPN), osteonectin (ON), and osteocalcin (OCN), providing a template for mineralization [25,26]. In addition, osteocytes regulate homeostasis via the Wnt signaling pathway, which includes the canonical β-catenin-dependent pathway and the non-canonical pathway. In the canonical pathway, Wnt ligands bind to frizzled (FZD) receptors and low-density lipoprotein receptor-related protein 5/6 (LRP5/6), recruiting dickkopf-1 (Dkk-1) to inhibit β-catenin degradation, allowing it to translocate to the nucleus and activate genes that regulate OBs and OCs [27,28,29,30]. OBs arrive at the bone matrix, and they begin depositing minerals and subsequently mature into osteocytes, which are embedded in the cavities of trabecular bone and cortical bone [23]. RANKL secreted by osteocytes and OBs bind to its receptor RANK of OC progenitors to differentiate into OCs but is also regulated by OPG, which inhibits excessive count or activity of OCs [31]. Therefore, OPG inhibit the binding of RANKL to RANK of OC progenitors, thereby suppressing the differentiation and activity of OCs [32].

**Figure 1:**
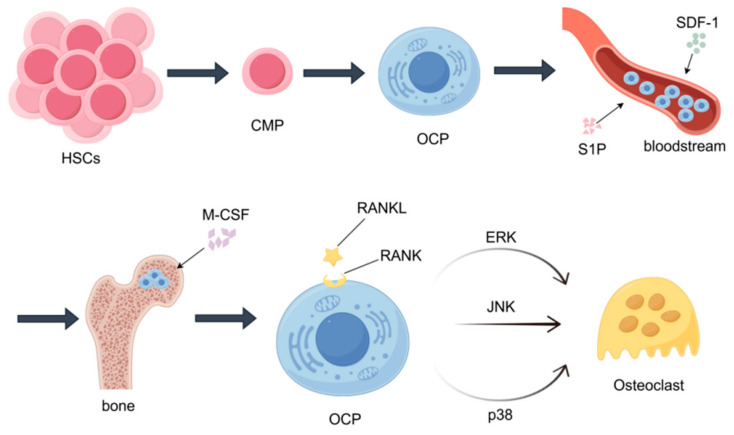
**Osteoclastogenesis.** HSCs develop into osteoclast precursors through CMPs and are recruited to the bone remodeling units through the action of S1P and SDF-1. The stimulation of M-CSF promotes the proliferation of osteoclast precursors and induces the binding of its surface receptor RANK to RANKL, activating the three classical MAPK signaling pathways of ERK, JNK, and p38, thereby promoting the differentiation and maturation of OCs.

In Figure 1, hematopoietic stem cells (HSCs) differentiate into common myeloid progenitors (CMPs), which further develop into osteoclast precursors. Osteoclast precursors are recruited to the bone remodeling unit via sphingosine-1-phosphate (S1P) and stromal cell-derived factor 1 (SDF-1), where macrophage colony-stimulating factor (M-CSF) promotes their survival and proliferation and the expression of RANK [33]. The binding of RANKL to RANK activates downstream mitogen-activated protein kinase (MAPK) signaling pathways (including ERK, JNK, and p38), leading to the activation of the transcription factors c-Fos and nuclear factor of activated T-cells cytoplasmic 1 (NFATc1), which drive osteoclast-specific gene expression. This cell–cell fusion process is highly energy-dependent, requiring substantial mitochondrial ATP to support cytoskeletal rearrangement and membrane remodeling. Next, the cells continue to differentiate and mature after fusion is complete, undergoing polarization to form the characteristic sealing zone and highly folded, invaginated ruffled border [34,35,36,37]. Ultimately, the mature OCs fuses into a large multinucleated cell, facing even greater metabolic demands to form its sealing zone and ruffled border, with the latter being a specialized structure for massive vesicular trafficking and acid secretion [3,37].

Therefore, the signaling pathways that orchestrate this energy-demanding differentiation process, particularly the “RANKL/RANK/NFATc1” axis, have become prime therapeutic targets for diseases involving excessive bone resorption [38]. The signaling cascade recruits TNF receptor-associated factor 6 (TRAF6) and activates IκB kinase (IKK) after RANKL–RANK binding, leading to nuclear factor kappa-B (NF-κB) nuclear translocation, which cooperates with c-Fos to induce NFATc1, the master transcriptional regulator of osteoclastogenesis [39]. In addition, pharmacological interventions targeting this axis have been extensively validated. For example, the natural decoy receptor OPG and its recombinant derivative RANK-Fc act as potent RANKL antagonists, effectively preventing RANKL-RANK binding [40,41]. Recently, the natural small-molecule compound Xuetongsu has been shown to directly target RANKL and suppress the “RANKL/RANK/NFATc1” axis, thereby inhibiting osteoclast differentiation and bone resorption [42]. These findings highlight that this axis is not only conserved across species but also readily druggable; chicken RANKL has been demonstrated to be functional and critical to chicken osteoclastogenesis and bone resorption [9,43]. This provides a strong theoretical basis for exploring therapeutic modulation of this pathway in bone disorders in layer chickens, particularly in the physiological importance of OC-driven medullary bone remodeling.

In addition, bone remodeling also maintains a dynamic balance between bone resorption and bone formation during the laying period of layer chickens. Rapidly growing layer chickens have a high demand of Ca during the laying period and have developed a unique medullary bone. Especially during sexual maturity, estrogen levels rise, and estradiol can stimulate the activity of OCs, promoting the deposition of collagen, non-collagenous proteins and a lipid matrix and then mineralizes to form medullary bone, leading to the transformation of OBs from forming structural bone to producing a large amount of medullary bone [5,14]. Furthermore, bone remodeling persists during egg-laying in layer chickens, and the rapid deposition and resorption of Ca require substantial energy [44,45]. In addition to being regulated by cytokines, the energy metabolism of the OCs themselves also plays a significant role in bone remodeling [46,47]. Importantly, the differentiation and maturation of OCs primarily depend on an adequate supply of glucose, fatty acids and amino acids, which provide ATP and biosynthetic substrates through glycolysis and mitochondrial OXPHOS [48,49,50,51]. Furthermore, the mitochondria of OCs play an important role in bone homeostasis through ROS-mediated redox signaling and apoptosis, and excessive mitochondrial ROS in OCs can lead to enhanced activity of OCs, thereby contributing to bone diseases such as OP [52].

## 3. Energy Metabolic Reprogramming of OCs in Layer Chickens

OCs are multinucleated, giant cells formed through the fusion of bone marrow mesenchymal stem cells (BMSCs) and bone marrow monocytes/macrophages (BMMs) and are crucial to bone resorption and degradation of bone matrix in dynamic bone remodeling [48]. The differentiation of OCs involves and activates metabolic programs, and the energy generated by metabolic reprogramming not only supports phenotypic changes from osteoclast precursor to multinucleated OCs but also facilitates bone resorption in mature OCs responsible for terminal differentiation [4,53]. As mentioned above, OXPHOS has been studied as the major metabolic pathway to fulfill the energy requirements of OCs, but all energy-related metabolic pathways are closely interconnected [54,55]. Next, the following sections summarize the current understanding of the metabolic reprogramming of OCs, but the relevance of these mechanisms likely differs by species. Therefore, the framework proposed here may be more directly applicable to regulating the activity of OCs through medullary bone turnover and eggshell-associated Ca mobilization in layer chickens. Although the evidence is limited, this framework can provide references for existing evidence of bone metabolic dysfunction in laying chickens or poultry.

### 3.1. Mitochondrial Dynamic Biogenesis of Osteoclastogenesis

RANKL-induced osteoclastogenesis promotes an increase in the size and the quantity of mitochondria within OCs, indicating that mature OCs contain abundant mitochondria [56]. Mitochondria play a prominent role in the dynamic equilibrium of energy within OCs and are one of the important organelles [54,57,58]. However, mitochondrial dysfunction causes the imbalance of energy metabolism within the differentiation and bone resorption function of OCs under pathological conditions. The regulation of mitochondrial shape and size are two important factors involved in mitochondrial dynamics, and they change mitochondrial shape and maintain its function through continuous division and fusion [56,59]. The major regulated mitochondrial fission proteins, i.e., dynamin-related protein 1 (DRP1) and mitochondrial fusion protein 1/2 (MFN1/2), are recruited to the mitochondrial membrane [60,61]. DRP1 contracts and cleaves the mitochondrial membrane through a mechanism dependent on GTP hydrolysis, while MNF1/2 mediates the fusion of the outer mitochondrial membrane [62]. Optic nerve atrophy protein 1 (OPA1) controls mitochondrial fusion bioenergetics and cristae integrity and is involved in mitochondrial inner membrane fusion [61]. Thus, when the balance of fission and fusion is disrupted, the mitochondrial quality control system is disrupted, resulting in mitochondrial dysfunction and cell death, which ultimately leads to tissue damage [56].

In addition, the differentiation and maturation of OCs are closely related to mitochondrial biosynthesis and function, such as PGC-1β, peroxisome proliferator-activated receptor gamma (PPAR-γ), and ERR α, which are very important in this process. Mitochondrial biogenesis in OCs involves both PGC-1β-dependent and -independent pathways, and PGC-1β may also regulate osteoclastogenesis through other unknown factors. Whether mitochondrial biogenesis is a prerequisite for osteoclast differentiation and function remains unclear. In the context of poultry, particularly layer chickens, emerging evidence has begun to elucidate the regulatory roles of mitochondrial dynamics-related proteins in differentiation and function of OCs. Studies have demonstrated that mitochondrial fission protein DRP1 and fusion proteins MFN1/2 and OPA1 are actively involved in the modulation of osteoclastogenesis in poultry [63,64,65]. For instance, transcriptomic analysis of osteoclastic differentiation in estrogen-deficient pullets revealed significant variations in genes associated with osteoclastic differentiation and mitochondrial function [66]. Exposure to environmental stressors such as ammonia (NH_3_) in broiler models was shown to activate mitochondrial fission-related genes (*drp1* and *mff*) while suppressing fusion-related genes (*opa1*, *mfn1*, and *mfn2*), leading to mitochondrial dysfunction and subsequent tissue damage [63]. Similarly, ochratoxin A exposure in broiler liver altered the expression of *drp1*, *mff*, *opa1*, *mfn1*, and *mfn2* at both mRNA and protein levels, indicating that mitochondrial dynamic imbalance is a common pathogenic mechanism in poultry [65]. High dietary copper was also found to disrupt mitochondrial dynamics in broiler spleen by upregulating DRP1 and downregulating MFN1, MFN2, and OPA1 [64]. Furthermore, selenomethionine was reported to alleviate mitochondrial dynamics imbalance via the toll-like receptor 4 (TLR4)/receptor-interacting serine/threonine-protein kinase 3 (RIPK3)/DRP1 signaling pathway in laying chickens, underscoring the therapeutic potential of targeting these proteins in poultry bone metabolic disorders [67].

Collectively, these findings indicate that the regulatory network of mitochondrial fission and fusion proteins is conserved across poultry and mammalian OCs, and that disruption of this network contributes to mitochondrial dysfunction and pathological bone resorption in poultry. However, direct evidence specifically linking these mitochondrial dynamic regulators to osteoclastic differentiation and bone-resorbing function in laying chickens remains limited. Further investigation is warranted to elucidate the precise molecular mechanisms by which DRP1, MFN1/2, and OPA1 modulate osteoclastogenesis in the context of laying chicken OP and to explore their potential as therapeutic targets for preventing bone loss in commercial poultry production.

### 3.2. OXPHOS of Osteoclastogenesis

During RANKL-induced osteoclastogenesis, mitochondrial biogenesis is markedly upregulated, accompanied by increased electron (e^−^) transport chain (ETC) enzyme activity, elevated oxygen consumption, and enhanced ATP production [48]. These coordinated metabolic adaptations indicate that the energy required for osteoclast differentiation is primarily derived from mitochondrial OXPHOS [63,68]. The transcription factor MYC, acting downstream of RANKL signaling, induces the expression of ERRα and genes encoding the ETC components localized in the inner mitochondrial membrane. Through the “MYC/ERRα/PGC-1β” axis, RANKL systematically promotes mitochondrial biogenesis and heme metabolism while upregulating the expression of genes involved in the tricarboxylic acid cycle (TCA) and OXPHOS, thereby enhancing mitochondrial respiratory capacity and membrane potential [68,69,70]. The ETC complexes, which comprise multiple polypeptides and prosthetic groups, function primarily to transfer electrons from respiratory coenzymes to oxygen. Through the functional coupling of cytochrome c and coenzyme Q (CoQ), these complexes harness the energy potential generated by e^−^ flux to synthesize ATP. Pharmacological inhibition of the ETC suppresses complex I activity, thereby impairing the formation and differentiation of OCs. Similarly, disruption of OXPHOS in OCs leads to alters the skeletal phenotype and reduces the number of OCs.

**Figure 2:**
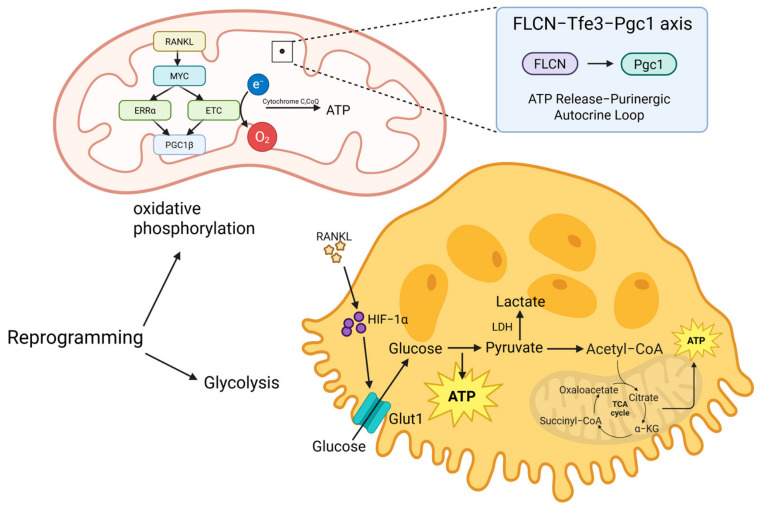
**Schematic depiction of metabolic reprogramming of OCs**. RANKL-driven MYC regulates ERRα and mitochondrial respiratory chain components, enabling e^−^ transfer through cytochrome c and CoQ to sustain ATP production. In parallel, FLCN deficiency shifts the metabolic landscape by activating the “Tfe3/Pgc1” axis, which boosts both OXPHOS and purine nucleotide synthesis, thereby establishing a self-amplifying purinergic signaling loop via extracellular ATP.

In contrast to differentiating osteoclasts, mature bone-resorbing OCs contain fewer mitochondria. When OXPHOS declines, these cells undergo a switch to glycolytic metabolism, and bone resorption is paradoxically enhanced [6,48,71]. This metabolic transition from OXPHOS to glycolysis in mature OCs is not merely a substitution of energy source but represents a functional adaption to the bone-resorbing microenvironment. First, the resorption lacuna constitutes a severely hypoxic niche that limits oxygen-dependent OXPHOS [72]. Second, glycolysis can generate ATP more rapidly than OXPHOS, meeting the sudden, high-energy demands of polarized membrane trafficking and proton pumping. Most importantly, the glycolytic end-product lactate is directly exported via monocarboxylate transporters (MCTs) to help acidify the resorption pit, a functional necessity independent of mitochondrial ATP production [4]. Additionally, loss of the tumor suppressor protein folliculin (FLCN) significantly enhances OXPHOS and purine metabolism through the “Flcn/Tfe3/Pgc1” axis, forming an ATP release–purinergic autocrine loop; that is, ATP itself acts as a signaling molecule that stimulates purinergic receptors on the cell surface, further driving the differentiation of OCs and consequently leading to bone loss (Figure 2) [73].

Whether the “MYC/ERRα/PGC-1β” axis and the OXPHOS-to-glycolysis metabolic switch operate similarly in OCs of poultry remains untested, but the experimental tools to address this question are readily available. Chicken RANKL has been cloned and functionally validated for inducing osteoclastogenesis from bone marrow cells, and osteoclast differentiation has been directly demonstrated during estrogen-induced medullary bone formation in Japanese quail [9,74]. Duck embryonic OCs have been successfully cultured and used to study the effects of RANKL, OPG, Ca, and P on osteoclast survival and activation [75]. These established OC systems could be directly employed to test whether the “MYC/ERRα/PGC-1β” pathway is functionally conserved across vertebrates. This question is most pertinent to layer chickens, whose OCs must cyclically resorb medullary bone to supply Ca for eggshell formation. In layer chickens, medullary bone formation and resorption are tightly coordinated with the 24 h egg-laying cycle, and OBs actively form medullary bone during the formative phase when the egg is in the magnum of the oviduct, whereas osteoclastic resorption is stimulated during the shell gland phase to mobilize Ca for eggshell calcification. Parathyroid hormone (PTH) has been shown to stimulate osteoclastic resorption in medullary bone, accompanied by increased succinate dehydrogenase (SDH) activity, a marker of mitochondrial respiratory capacity, and the development of ruffled borders [76]. Conversely, calcitonin directly inhibits osteoclastic bone resorption, decreasing both SDH and lactate dehydrogenase (LDH) activity and causing the disappearance of ruffled borders within one hour [77]. These hormonal responses suggest that mitochondrial metabolism in poultry medullary bone OCs is subject to acute regulation that mirrors the rapid Ca demands of eggshell formation. Furthermore, transcriptomic analyses in chickens have identified that OXPHOS pathway genes, including all annotated mitochondrial and nuclear-encoded OXPHOS genes, are differentially expressed in association with growth and metabolic processes, providing a molecular foundation for investigating how these pathways are regulated in laying hen osteoclasts during the medullary bone cycle [78].

### 3.3. Glycolysis of Osteoclastogenesis

Aerobic glycolysis in OCs is essential to their activation and bone resorption, yet it does not appear to be a prerequisite for osteoclastogenesis. Glycolytic enzymes convert glucose into two molecules of pyruvate, generating a net yield of two molecules per glucose. Under aerobic conditions, pyruvate can enter the TCA, fueling OXPHOS to generate large amounts of ATP. Alternatively, pyruvate is converted into lactate dehydrogenase (LDH) in a process that regenerates the oxidized nicotinamide adenine dinucleotide (NAD^+^) required to sustain ongoing glycolysis, albeit without additional ATP production [79].

HIF-1α activation in OCs is classically viewed as an oxygen-dependent response to hypoxia during osteoclastogenesis [80]. However, RANKL can also activate HIF-1α under normoxic conditions, revealing the existence of an oxygen-independent pathway. Thus, HIF-1α integrates both hypoxic and RANKL-derived signals, and its activation is not exclusively dependent on either factor alone [4]. HIF-1α upregulates the expression of the glucose transporter GLUT1 and various glycolytic enzymes after activation, thereby enhancing bone-resorptive capacity of OCs. Whether OCs in poultry undergo a similar HIF-1α-driven glycolytic shift during bone resorption and how this glycolytic program interfaces with OXPHOS to meet the bioenergetic demands of medullary bone mobilization remain open questions that could be addressed using existing duck or chicken OC cultures. Conversely, glucose deprivation or pharmacological inhibition of HIF-1α reduces GLUT1 and glycolytic gene expression, suppressing the formation and bone resorption of OCs [81]. Therefore, the “HIF-1α/GLUT1/glycolysis” axis could represent a target for modulating medullary bone resorption in layer chickens, for instance, by using dietary histone deacetylase (HDAC) inhibitors (e.g., butyrate) to suppress osteoclast glycolysis [82,83,84].

In addition, glycolytic intermediates have also been shown to affect osteoclast differentiation and activity. For instance, fructose-1,6-bisphosphate, when administered at high concentrations (100 µmol/L and 300 µmol/L) in vitro, inhibits RANKL-induced osteoclastogenesis and tartrate-resistant acid phosphatase (TRAP) activity by suppressing the NF-κB/NFATc1 signaling pathway [85]. LDH activity increases during RANKL-induced osteoclastogenesis and is responsible for converting pyruvate to lactate (Figure 2). Although lactate or pyruvate alone does not alter bone resorption, both metabolites can reverse the decrease in bone resorption caused by glycolytic inhibition with 2-deoxy-D-glucose (2-DG), indicating that lactate and pyruvate produced by glycolysis are important mediators of enhanced bone resorption [86]. Furthermore, monocarboxylate transporters (MCTs) export the lactate produced by increased glycolysis out of the cell. This not only prevents intracellular lactate accumulation and subsequent cellular damage, but the exported lactate also contributes directly to creating the acidic environment required for osteoclastic bone resorption, thereby promoting resorptive activity [6].

### 3.4. Regulation of OCs by ROS

ROS constitute a collective term for a class of oxygen-containing, chemically active molecules or free radicals, including superoxide anion radical (O_2_^−^), hydrogen peroxide (H_2_O_2_), organic hydroperoxide (ROOH), hydroxyl radical (−OH), and ozone (O_3_), among others. ROS are primarily generated in mitochondria through e^−^ escape from the electron transport chain and participate in both signal transduction and oxidative damage in living organisms. Nicotinamide adenine dinucleotide phosphate (NADPH) oxidase (NOX), a multi-component enzyme complex, has been identified as one of the key enzymatic sources of ROS [87].

It is crucial to recognize the dual nature of ROS signaling in the biology of OCs, a conceptual framework framed as the balance between oxidative eustress and oxidative distress. At low-to-moderate (physiological) concentrations, ROS act as essential second messengers, transiently activating pro-survival and pro-differentiation pathways. For instance, RANKL and M-CSF, the indispensable cytokines for osteoclast formation, increase ROS levels, which, in turn, potentiate osteoclast differentiation, activation, and survival. Importantly, this controlled redox signaling is a prerequisite for normal bone remodeling.

Conversely, excessive or sustained ROS production shifts this balance toward pathology. Supraphysiological ROS levels cause oxidative damage to cellular lipids, proteins, and DNA, a state known as “oxidative distress” [7,88]. High levels of ROS hyperactivate key signaling cascades such as NF-κB and MAPK, leading to the upregulation of the master transcription factor NFATc1 and downstream osteoclast-specific genes (e.g., cathepsin K and TRAP). This results in the formation of hyperactive, long-lived OCs that resorb bone at an accelerated rate, a key driver of bone loss in diseases like postmenopausal OP [89].

In addition, upon the binding of RANKL to its receptor RANK, tumor necrosis factor (TNF) receptor-associated factor 6 (TRAF6) is recruited, which subsequently activates multiple downstream signaling pathways [38]. Rac family small GTPase 1 (Rac1), a cytoplasmic component of the NOX complex, enables NOX1 within the complex to transfer electrons from NADPH to molecular oxygen, thereby generating ROS [87,90]. Furthermore, studies have indicated that RANKL can activate autophagy and promote the differentiation of OCs through ROS-mediated downregulation of HIF-1α [91]. It can also promote ROS production and the differentiation of OCs by degrading metallothionein 2 (MT2) protein through the Beclin1-dependent autophagy pathway [92]. ROS can also promote the maturation of OCs by activating key signaling pathways, such as MAPK, phosphoinositide 3-kinase (PI3K), and NF-κB, leading to the upregulation of downstream genes such as *ctsk*, *matrix metalloproteinase 9 (mmp9)*, and *nfstc1* [90]. This indicates that ROS and the differentiation of OCs mutually influence each other and are closely interconnected.

The antibody 121F caused the contraction of OCs in chickens, reduced TRAP activity, and inhibited bone resorption [93]. Mechanistically, 121F binds to a superoxide dismutase-related protein on the surface of OCs, blocking its protective function of scavenging superoxide. As a result, superoxide levels rise sharply due to inadequate clearance. This surge in superoxide then stimulates OCs to release more nitric oxide (NO), which will ultimately act as a “stop signal” to effectively inhibit the bone-resorbing activity of OCs [93]. The 121F antibody study demonstrates that the “superoxide–NO” axis can modulate bone resorption of OCs in layer chickens, representing the most direct evidence for redox regulation of the function of OCs. However, the study used a monoclonal antibody tool rather than investigating endogenous metabolic reprogramming, and the OCs were of chicken’s origin but were studied ex vivo.

### 3.5. Mitophagy Maintains Mitochondrial Quality and Supports the Maturation of OCs

Mitophagy exhibits dynamic temporal characteristics and plays an indispensable role in both differentiation and maturation of OCs [94]. In the early stage of RANKL-induced formation of OCs, the expression levels of several autophagy-related proteins such as autophagy-related 5 (ATG5), ATG7, and ATG12, together with microtubule-associated protein 1 light chain 3 II (LC3II), are significantly increased, accompanied by enhanced degradation of the autophagy adaptor sequestosome 1 (p62/SQSTM1). These observations collectively indicate that autophagy is robustly activated during the initial stages of osteoclastogenesis [69].

Aoki et al. [95] provided further evidence that autophagic activity is progressively enhanced throughout the differentiation and maturation of OCs and that this activity is positively correlated with the activity and survival of OCs. Mechanistically, when mitochondrial OXPHOS is upregulated, the small GTPase Ras homolog enriched in brain (Rheb) accumulates on the outer mitochondrial membrane, where it triggers mitophagy to selectively eliminate damaged mitochondria. Given that heightened OXPHOS activity generates large amounts of ROS and induces mitochondrial damage, mitophagy serves as a critical quality control mechanism that sustains mitochondrial homeostasis during osteoclast differentiation. Beyond enhancing ATP production by supplying intermediates to the mitochondrial TCA, autophagy preserves mitochondrial functional integrity through mitochondrial clearance. Therefore, mitophagy constitutes an essential regulatory mechanism for the differentiation and maturation of OCs.

In the context of poultry, particularly laying chickens, OC-mediated bone remodeling is of paramount importance due to the massive calcium demand imposed by eggshell formation [68]. In laying chickens, the intensive Ca mobilization from medullary and cortical bone during the laying cycle places extraordinary metabolic demands on OCs, which are mitochondria-rich cells that rely heavily on OXPHOS for energy production [96,97,98]. Notably, chicken OCs possess abundant mitochondria and exhibit high cytochrome c oxidase activity, a hallmark of oxidative metabolic capacity [99]. During RANKL-induced osteoclastogenesis in poultry species, mitochondrial biogenesis is significantly upregulated, accompanied by increased mitochondrial membrane potential and enhanced OXPHOS activity [96]. This metabolic reprogramming toward oxidative metabolism is essential to supporting the energetic demands of the fusion of OCs, ruffled border formation, and bone matrix degradation [54,71]. Furthermore, studies in chicken models have shown that modulators of the activity of OCs, such as OPG, exert their inhibitory effects in part through the activation of autophagy pathways, highlighting the interplay between autophagic regulation and osteoclast function in poultry species [11]. Given the high metabolic rate and oxidative stress burden in the OCs of laying chickens during peak production, mitophagy likely plays a particularly critical role in maintaining mitochondrial quality and preventing the accumulation of dysfunctional mitochondria that could compromise bone resorption efficiency and bone integrity.

## 4. The “Lysosome/Fe/Mitochondria” Axis Regulates Osteoclast Energy Metabolism

OCs maintain bone remodeling by resorbing and degrading the bone matrix, a process that is highly dependent on lysosomes [38,100]. Lysosomal acidification is a prerequisite for proper Fe handling, as it prevents the accumulation of toxic Fe ions and facilitates both the release of Fe^3+^ from the “transferrin receptor (TfR)–transferrin (Tf)–Fe” and the recycling of Fe^2+^ from ferritin. Intracellular free Fe^2+^ is highly toxic and must be either stored or utilized via divalent metal transporter 1 (DMT1). DMT1 expression increases during osteoclastogenesis and promotes the differentiation of OCs [101]. Thus, lysosome-mediated Fe transport plays a critical role in regulating the activity of OCs.

Beyond lysosomal function, Fe is indispensable for mitochondrial energy metabolism, contributing not only to OXPHOS but also to generation of mitochondrial ROS. Once transported into the mitochondria, Fe participates in the biosynthesis of heme and Fe-sulfur (S) clusters, which serve as essential cofactors for key enzymes in energy production. OCs must acquire sufficient Fe primarily through TfR1 to generate the energy required for bone resorption [102]. Fe can also generate ROS via the “Fenton reaction”, a redox reaction in which Fe^2+^ reacts with hydrogen peroxide (H_2_O_2_) to produce hydroxyl radicals (−OH) [103,104]. As a key component of the ETC, Fe facilitates electron transfer during OXPHOS in mitochondria, generating ROS that traverse the inner mitochondrial membrane. In the Fe^2+^ state, Fe can donate electrons, whereas in the Fe^3+^ state, it can accept electrons. This dual redox capability is crucial to mediating e^−^ transfer and ROS generation within mitochondrial ETC complexes I, II, and III [103,105].

The “lysosome/Fe/mitochondria” axis controls Fe transport and recycling between lysosomes and mitochondria in OCs. This axis supports mitochondrial energy metabolism and redox balance, including TfR1 (responsible for Fe uptake), divalent metal transporter 1 (DMT1; responsible for lysosomal Fe export), mitoferrin 2 (MFRN2; responsible for mitochondrial Fe acquisition), and transcription factor EB (TFEB; responsible for the transcriptional coordination of lysosomal and mitochondrial biogenesis) [8,106,107]. Disruption of the expression or function of any of these molecules through gene knockout or antibody inhibition leads to decreased bone resorption capacity or increased bone mass of OCs, via effects on pathways such as Ca signaling and NFATc1 activation. Furthermore, OCs from lysosomal or mitochondrial dysfunction models exhibit resorption defects, whereas macrophages are relatively unaffected, further demonstrating that OCs are highly sensitive to perturbations of this axis [8,108].

In bone metabolism, dysregulation of the “lysosome/Fe/mitochondria” axis extends beyond simple metabolic failure and can trigger ferroptosis. Ferroptosis, an Fe-dependent form of regulated cell death, is characterized by glutathione peroxidase 4 (GPX4) inactivation, glutathione depletion, and Fe-dependent accumulation of lipid peroxides [109]. Fe overload promotes ROS generation via the “Fenton reaction”, inactivates GPX4, and triggers a lipid peroxidation cascade. The oxidative stress microenvironment activates the NF-κB signaling pathway, leading to upregulation of TfR1 and enhanced Fe uptake in osteoclast precursors, further amplifying ROS generation and establishing a vicious cycle of “oxidative stress–ferroptosis–cell death” [110]. Recent evidence links ferroptosis to the pathogenesis of OP, and ferroptosis disrupts the balance between OB-mediated bone formation and OC-driven bone resorption. Fe overload enhances the activity of OCs and accelerates bone resorption, while ferroptosis in OBs reduces bone formation. This suggests that stabilizing mitochondrial redox balance and inhibiting Fe-dependent lipid peroxidation could represent a novel therapeutic strategy for bone loss disorders [111].

In addition to ferroptosis mediated by the “lysosome/Fe/mitochondria” axis, post-transcriptional regulation also plays an important role. A recent study demonstrated that in a titanium nanoparticle-induced osteolysis model, YTHDF1, a key m^6^A modification reader protein, is transcriptionally activated by TCF4. Activated TCF4 promotes YTHDF1 transcription, which in turn enhances the translation efficiency of GPX4 and solute carrier family 7 member 11 (SLC7A11), thereby effectively suppressing OB ferroptosis and alleviating osteolysis. The study reveals a novel mechanism by which the “Wnt/β-catenin/TCF4/YTHDF1” axis regulates ferroptosis in bone cells, providing new insights into the pathogenesis of bone metabolic disorders and identifying potential therapeutic targets [112].

The “lysosome/Fe/mitochondria” axis has not been investigated in any avian species. Fe metabolism in layer chickens is of particular interest because egg production imposes a high demand for Fe, which is deposited in egg yolk, and the interplay between systemic Fe homeostasis and bone health remains unexplored. Dietary Fe supplementation or restriction could theoretically modulate the activity of OCs through this axis, but the risk of inducing ferroptosis in OBs must be carefully considered. Given the complete absence of any avian data, the “lysosome/Fe/mitochondria” axis represents a high-priority area for exploratory research using poultry osteoclast models.

## 5. Limitations and Future Perspectives

Current knowledge on poultry bone diseases disproportionately focuses on chondrocytes for several practical reasons. The high incidence and substantial economic burden of TD and femoral head necrosis (FHN) have naturally directed investigative attention toward the growth plate cartilage as an intuitive lesion site. Furthermore, isolating and culturing primary poultry OCs requires specialized protocols that remain less standardized than those for mammalian OCs. Nevertheless, it is important to recognize that the model systems of poultry OCs do exist, and they have been successfully established and validated in chickens, ducks, Japanese quail, and other species. Such studies have demonstrated the differentiation, activation, and bone resorption of OCs and responsiveness to pharmacological and environmental stimuli (Table 1). The critical limitation, however, is that none of these established systems of poultry OCs have been applied to investigate mitochondrial energy metabolism, metabolic reprogramming, the “lysosome/Fe/mitochondria” axis, etc. This constitutes a specific and addressable research gap rather than an insurmountable methodological barrier. Until Seahorse flux analysis, mitochondrial imaging, and metabolomic profiling are performed on poultry OCs, the field will continue to rely on extrapolation from mammalian data, but the tools to move beyond extrapolation are now available.

A critical biological caveat that must guide future research is the fundamentally different bone pathophysiology of broilers versus layer chickens. The bone disorders of broiler are mainly caused by defects in endochondral ossification, dysfunction of chondrocytes, vascular damage, and biomechanical overload [7,11,17], while OC-mediated bone resorption is not considered a primary pathogenic factor of these diseases. Therefore, the framework of metabolic reprogramming in OCs proposed in this review may have limited direct relevance to the bone health of layer chickens.

## 6. Conclusions and Perspectives

There is no evidence of mitochondrial biology of OCs unique to layer chickens, but a key knowledge gap that must be bridged before metabolism of OCs can be used as a therapeutically targeted has been identified. We summarize the current state of knowledge across three tiers. The differentiation and bone resorption of OCs are tightly coupled to mitochondrial energy metabolism. The OXPHOS-to-glycolysis metabolic switch, regulated by the “MYC/ERRα/PGC-1β” and “RANKL/HIF-1α” axes, constitutes a functional prerequisite for bone resorption. ROS serve dual roles as signaling molecules (eustress) and pathological drivers (distress), with sirtuin 3 (SIRT3)-mediated mitochondrial quality control as a key protective mechanism. In addition, mitophagy and the “lysosome/Fe/mitochondria” axis represent emerging regulatory nodes, and their dysregulation can drive ferroptosis and pathological bone loss.

In vitro culture systems have been established in chickens, ducks, quail or other species of poultry, demonstrating that the differentiation, activation, and bone resorption of OCs can be studied in vitro. Osteoclastogenesis has been directly observed during medullary bone formation in quail, and poultry OCs respond to redox modulation and pharmacological stimuli. The transcriptomic profiling of laying chicken bone tissue has revealed metabolic pathway alterations associated with bone disorders, while broiler chondrocytes exhibit glycolytic reprogramming and mitochondrial dysfunction in bone lesions. However, none of these studies directly address mitochondrial metabolic reprogramming in poultry OCs.

The translational framework presented here generates several testable hypotheses. The OXPHOS-to-glycolysis switch is conserved in OCs of layer chickens during medullary bone resorption; the “lysosome/Fe/mitochondria” axis regulates the activity of OCs in layer chickens subjected to high Fe demands for egg production; or dietary short-chain fatty acids (SCFAs) and organic trace minerals can modulate mitochondrial function of OCs in poultry. Testing these hypotheses is now technically feasible using established avian osteoclast culture systems, specifically: (1) Seahorse metabolic flux analysis of chicken OCs can directly measure OXPHOS and glycolytic rates; (2) CRISPR-Cas9 gene editing in chicken bone marrow-derived osteoclast precursors can validate candidate metabolic genes; (3) comparative metabolomics of layer chickens versus broiler OCs can reveal poultry-specific metabolic adaptations. The ultimate goal is discovering poultry-specific metabolic adaptations that have evolved under the extreme physiological demands of rapid growth and sustained egg production. Therefore, achieving this will require a definitive shift toward applying existing avian osteoclast tools to mitochondrial biology, which will transform our ability to prevent skeletal disorders in commercial flocks through precisely targeted interventions.

## Figures and Tables

**Table 1:** Existing model systems in poultry OCs.

Species	Cell/Tissue Source	Induction Method	Validated Endpoints	Reference
Chicken	Bone marrow cells	Recombinant chicken RANKL	Osteoclast differentiation, bone resorption, and direct activation of mature OCs	Wang et al., 2008 [9]
Chicken	Bone marrow/spleen	RANKL + M-CSF	Osteoclast purification via monoclonal antibody (121F) coupled to magnetic beads	Collin-Osdoby et al., 1998 [93]
Muscovy duck	Embryonic bone marrow	RANKL, OPG, Ca^2+^, and PO_4_^3−^	Osteoclast survival, activation, and apoptosis	Gu et al., 2009 [75]
Gaoyou duck	Embryonic bone marrow	RANKL + M-CSF	Osteoclast differentiation, activation, and apoptosis; OPG effects	Fu et al., 2013 [113]
Gaoyou duck	Embryonic bone marrow	RANKL + M-CSF + inhibitors	Flavonoid-mediated inhibition of osteoclast differentiation	Fu et al., 2019 [114]
Duck	Embryonic bone marrow	Cadmium exposure	Osteoclast differentiation via P2 × 7/PI3K/AKT pathway	Ma et al., 2021 [115]
Duck	Embryonic bone marrow	Cadmium + melatonin	Osteoclast/osteoblast differentiation and pyroptosis	Ma et al., 2024 [116]
Japanese quail	Bone marrow cells	Estrogen (in vivo)	Osteoclastogenesis during medullary bone formation	Hiyama et al., 2019 [74]
Japanese quail	In vivo (male)	Estrogen	Medullary bone formation and osteoclast activity (histomorphometry)	van de Velde et al., 1985 [117]

## Data Availability

No datasets were generated or analyzed in this study.

## Abbreviations

The following abbreviations are used in this manuscript:

**Abbreviation**	**Full name**
2-DG	2-deoxy-D-glucose
ALP	alkaline phosphatase
ANT	adenine nucleotide translocase
ATG5	autophagy-related 5
ATP	adenosine triphosphate
ATP6v0d2	ATPase H^+^ transporting V0 subunit d2
AVUCP	avian uncoupling protein
BCO	bacterial chondronecrosis with osteomyelitis
BMMs	bone marrow monocytes/macrophages
BMSCs	bone marrow mesenchymal stem cells
BRU	bone remodeling unit
BSP	bone sialoprotein
Ca	calcium
ClC-7	chloride voltage-gated channel 7
CMPs	common myeloid progenitors
COL1	type I collagen
CoQ	coenzyme Q
COX4	cytochrome c oxidase subunit IV
COX5A	cytochrome c oxidase subunit 5A
CPs	cysteine proteases
CRISPR	clustered regularly interspaced short palindromic repeats
CTSK	cathepsin K
DC-STAMP	dendritic cell-specific transmembrane protein
Dkk-1	dickkopf-1
DMT1	divalent metal transporter 1
DRP1	dynamin-related protein 1
ECM	extracellular matrix
ERK	extracellular signal-regulated kinase
ERRα	estrogen-related receptor alpha
ETC	electron transport chain
e^−^	electron
Fe	ferrum
Fe^2+^	ferrous iron
Fe^3+^	ferric iron
FHN	femoral head necrosis
FLCN	folliculin
FZD	frizzled
Glut1	glucose transporter 1
GPX4	glutathione peroxidase 4
HDAC	histone deacetylases
HIF-1α	hypoxia inducible factor-1 alpha
HIFs	hypoxia-inducible factors
HSCs	hematopoietic stem cells
H_2_O_2_	hydrogen peroxide
JNK	c-Jun N-terminal kinase
LC3II	microtubule-associated protein 1 light chain 3 II
LDH	lactate dehydrogenase
LRP5/6	low-density lipoprotein receptor-related protein 5/6
Mab	monoclonal antibody
MAPK	mitogen-activated protein kinase
M-CSF	macrophage colony-stimulating factor
MCTs	monocarboxylate transporters
MFRN2	mitoferrin 2
MMP9	matrix metalloproteinase 9
MMPs	matrix metalloproteinases
MFN1/2	mitochondrial fusion protein 1/2
MT2	metallothionein 2
MYC	myelocytomatosis oncogene
NADPH	nicotinamide adenine dinucleotide phosphate
NAD^+^	oxidized nicotinamide adenine dinucleotide
NFATc1	nuclear factor of activated T-cells cytoplasmic 1
NF-κB	nuclear factor kappa-B
NH_3_	ammonia
NO	nitric oxide
NOX	NADPH oxidase
NOX1	NADPH oxidase 1
OBs	osteoblasts
OCN	osteocalcin
OCs	osteoclasts
OC-STAMP	osteoclast-stimulating transmembrane protein
−OH	hydroxyl radical
ON	osteonectin
OPA1	Optic nerve atrophy protein 1
OPG	osteoprotegerin
OPN	osteopontin
OXPHOS	oxidative phosphorylation
O_2_^−^	superoxide anion radical
O_3_	ozone
P	phosphorus
p38	p38 MAPK
p62/SQSTM1	sequestosome 1
PTH	parathyroid hormone
PGC-1β	peroxisome proliferator-activated receptor gamma coactivator 1-beta
PI3K	phosphoinositide 3-kinase
Plexin-B1	plexin-B1 (receptor on OBs)
PPAR-γ	peroxisome proliferator-activated receptor gamma
Rac1	Rac family small GTPase 1
RANK	receptor activator of nuclear factor-κB
RANKL	receptor activator of nuclear factor-κB ligand
Rheb	Ras homolog enriched in brain
RIPK3	receptor-interacting serine/threonine-protein kinase 3
ROOH	organic hydroperoxide
ROS	reactive oxygen species
Runx2	runt-related transcription factor 2
S	sulfur
S1P	sphingosine-1-phosphate
SCFAs	short-chain fatty acids
SDF-1	stromal cell-derived factor 1
SDH	succinate dehydrogenase
SIRT3	sirtuin 3
SLC7A11	solute carrier family 7 member 11
Sox9	SRY (Sex-determining Region Y)-box transcription factor 9
SRY	Sex-determining Region Y
TCA	tricarboxylic acid cycle
TD	tibial dyschondroplasia
Tf	transferrin
TFEB	transcription factor EB
TfR	transferrin receptor
TfR1/Tfr1	transferrin receptor 1
TLR4	toll-like receptor 4
TNF	tumor necrosis factor
TRACP	tartrate-resistant acid phosphatase
TRAF6	TNF receptor-associated factor 6
TRAP	tartrate-resistant acid phosphatase
V-ATPase	vacuolar-type ATPase
VDAC3	voltage-dependent anion channel 3
Wnt	Wingless-related integration site
